# Predicting and managing primary and secondary non-response to rituximab using B-cell biomarkers in systemic lupus erythematosus

**DOI:** 10.1136/annrheumdis-2017-211191

**Published:** 2017-07-06

**Authors:** Md Yuzaiful Md Yusof, Daniel Shaw, Yasser M El-Sherbiny, Emma Dunn, Andy C Rawstron, Paul Emery, Edward M Vital

**Affiliations:** 1 Leeds Institute of Rheumatic and Musculoskeletal Medicine, University of Leeds, Chapel Allerton Hospital, Chapeltown Road, Leeds, UK; 2 NIHR Leeds Biomedical Research Centre, Leeds Teaching Hospitals NHS Trust, Leeds, UK; 3 Department of Clinical Pathology, Faculty of Medicine, Mansoura University, Egypt; 4 Department of Nephrology, St James’ University Hospital, Leeds Teaching Hospitals NHS Trust, Leeds, UK; 5 Haematological Malignancy Diagnostic Service, Leeds Teaching Hospitals NHS Trust, Leeds, UK

**Keywords:** treatment, B-cells, DMARDs (biologic), systemic lupus erythematosus

## Abstract

**Objective:**

To assess factors associated with primary and secondary non-response to rituximab in systemic lupus erythematosus (SLE) and evaluate management of secondary non-depletion non-response (2NDNR).

**Methods:**

125 patients with SLE treated with rituximab over 12 years were studied prospectively. A major clinical response was defined as improvement of all active British Isles Lupus Assessment Group (BILAG)-2004 domains to grade C/better and no A/B flare. Partial responders were defined by one persistent BILAG B. B-cell subsets were measured using highly sensitive flow cytometry. Patients with 2NDNR, defined by infusion reaction and defective depletion, were treated with ocrelizumab or ofatumumab.

**Results:**

117 patients had evaluable data. In cycle 1 (C1), 96/117 (82%) achieved BILAG response (major=50%, partial=32%). In multivariable analysis, younger age (OR 0.97, 95% CI 0.94 to 1.00) and B-cell depletion at 6 weeks (OR 3.22, 95% CI 1.24 to 8.33) increased the odds of major response. Complete depletion was predicted by normal complement and lower pre-rituximab plasmablasts and was not associated with increased serious infection post-rituximab. Seventy-seven (with data on 72) C1 responders were retreated on clinical relapse. Of these, 61/72 (85%) responded in cycle 2 (C2). Of the 11 C2 non-responders, nine met 2NDNR criteria (incidence=12%) and tested positive for anti-rituximab antibodies. Lack of concomitant immunosuppressant and higher pre-rituximab plasmablasts predicted 2NDNR. Five were switched to ocrelizumab/ofatumumab, and all depleted and responded.

**Conclusion:**

Treatment with anti-CD20 agents can be guided by B-cell monitoring and should aim to achieve complete depletion. 2NDNR is associated with anti-rituximab antibodies, and switching to humanised agents restores depletion and response. In SLE, alternative anti-CD20 antibodies may be more consistently effective.

## Introduction

Rituximab, a chimeric anti-CD20 monoclonal antibody (mAb) remains an important treatment option for moderate to severe systemic lupus erythematosus (SLE). A high degree of efficacy of rituximab across a range of lupus manifestations has been reported in open-label studies from single-centre series,^1–3^ multicentre registries^4–6^ and a systematic review of off-label use.^7^ Despite the success of these series, two phase III randomised placebo-controlled trials in non-renal lupus^8^ and renal lupus^9^ failed to meet their primary end-points. The discrepancy between the randomised trials and real-world evidence has been attributed to aspects of trial design including choice of end-points, the use of an active comparator, inclusion criteria and low statistical power.^10^

Nevertheless, there are also mechanistic reasons for the failure of rituximab in clinical trials in SLE. B-cell killing by rituximab appeared less efficient in SLE than rheumatoid arthritis (RA)^11^ due to internalisation through interaction with FcγRIIb resulting in reduced effector activity^12^ and pathogenic lupus autoantibodies that were produced by long-lived plasma cells.^13 14^ Using highly sensitive flow cytometry (HSFC), a protocol that was optimised for the detection of plasmablasts, we discovered that the depth of B-cell depletion predicted response in RA^15^ and SLE.^2^ Similar studies as well as identifying other clinical predictors of response to rituximab in SLE are needed to optimise its use and to help design trials of alternative B-cell depleting strategies.

B-cell depletion therapy with rituximab is transient. Some patients with initial good response experience relapse after B-cell repopulation (although with a variable interval). In our published discovery cohort, we showed a bimodal pattern of relapse. Earlier relapse requiring rituximab retreatment was predicted by a plasmablast count of >0.0008×10^9^/L at 6 months (the time of initial clinical response).^2^ Patients with lower plasmablasts at 6 months had sustained response without retreatment. Validation of this as a biomarker is therefore needed to determine whether HSFC can be used in clinical practice to guide retreatment decisions.

Repeat treatment with rituximab is effective.^1^ However, we observed cases of patients with SLE who had previously depleted and responded well to rituximab but subsequently developed (1) a severe infusion reaction >24 hours during the second infusion of a cycle, (2) failure to deplete CD20+ (naïve and memory) B-cells and (3) clinical non-response during repeat cycles. We called this phenomenon secondary non-depletion and non-response (2NDNR), which was suggestive of immunogenicity to rituximab and could be overcome by alternative anti-CD20 mAbs, particularly humanised. Therefore, the aims of the study were to assess factors predicting primary and secondary non-response to rituximab in SLE including validation of B-cell depletion and to evaluate management of 2NDNR using alternative anti-CD20 agents.

## Methods

### Patients and design

A prospective observational study was conducted of all patients with moderate to severe SLE who were treated with rituximab in Leeds between January 2004 and July 2016. Inclusion criteria included (1) adults (>16 years old); (2) fulfilling the revised 1997 American College of Rheumatology classification for SLE^16^ and (3) at least 6 months follow-up post-rituximab.

### Treatment protocol

All patients received a first cycle of therapy consisting of 100 mg of methylprednisolone and 1000 mg of rituximab given intravenously on days 1 and 14. Further cycles of the same regimen were repeated on clinical relapse (defined below).

Of those who met 2NDNR criteria, their treatment was switched from rituximab to humanised anti-CD20 mAbs either by using (1) 2×1000 mg ocrelizumab (compassionate use from Roche UK) or (2) 2×700 mg ofatumumab (individual funding request to NHS England).

### Clinical data and outcomes

Disease activity was assessed using the British Isles Lupus Assessment Group (BILAG-2004)^17^ at baseline and every 3 months thereafter. Clinical responses at 6 months were determined as following: (1) major clinical response=improvement of all domains rated A/B to grade C/better and no A/B flare between baseline and 6 months; (2) partial clinical response=maximum of 1 domain with a persistent grade B with improvement in all other domains and no A or B flare and (3) non-response=those not meeting the criteria for major or partial clinical response. Relapse was defined as a new grade A or recurrence of ≥1 grade B following either major/partial clinical response at 6 months. Global BILAG score was calculated as follows: grade A=12, grade B=8, grade C=1 and grades D and E=0.^18^

### Laboratory assessments

Peripheral blood B-cell subsets (naïve, memory B-cells and plasmablasts) were measured using HSFC as previously described^15^ at baseline, 6 months and every 6 months without knowledge of clinical status other than time since rituximab. Complete B-cell depletion was defined as counts <0.0001×10^9^/L and repopulation as ≥0.0001×10^9^/L.

Anti-dsDNA antibody titres were measured by ELISA until July 2012 and Bioplex 2200 Immunoassay (after July 2012). Complement levels (C3 and C4) and total serum immunoglobulin titres were measured by nephelometry.

Anti-rituximab antibodies were tested on a subset of patients with 2NDNR using the Promonitor® Anti-Rituximab ELISA according to the manufacturer’s instructions and compared these concentrations to those with continued response to rituximab. A positive test (as determined by the manufacturer) was concentration >140 AU/mL.

### Safety

Serious infections were recorded irrespective of suspected association with SLE and/or therapy. These were infections that resulted in hospitalisation for >24 hours or required intravenous antibiotics. Details about other safety assessment can be found in online supplementary files.

### Statistical analysis

Descriptive statistics were summarised using mean with SD or median with IQR for continuous variables and proportion for categorical variables. Multiple imputation was used for missing data. Multivariable analyses were performed using logistic regression after checking for multicollinearity. The significance of the association between categorical variables was tested by Fisher’s exact test, while for continuous variables using Mann-Whitney U test. Receiver operator curves (ROCs) were used to measure sensitivity and specificity of optimal thresholds for investigations predicting time-to-clinical relapse.

All statistical analysis was performed using Stata V.13.1 and Graph Pad Prism V.6.01 for Windows.

## Results

### Patient characteristics

Of 125 patients with SLE who were treated with rituximab at our unit, 117 patients with evaluable data at 6 months were studied. Baseline characteristics are described in table 1. One hundred and twelve (96%) had refractory and active disease as defined by BILAG ≥1A score and/or ≥2B scores. The remaining five had BILAG B in one domain only but was refractory to other conventional therapies as well as on maintenance with oral prednisolone ≥10 mg daily. Total follow-up was 492 patient-years.

**Table 1:** Baseline characteristics of the 117 patients with SLE treated with rituximab

Age at first RTX infusion, median (IQR) years	39 (26–52)	
No. female patient (%)	109 (93)	
Ethnicity, N (%)		
Caucasian	80 (68)	
Afro-Caribbean	11 (10)	
South Asian	20 (17)	
Other	6 (5)	
SLE disease duration at first RTX, median (IQR) years	6 (2–11)	
Positive ANA at diagnosis, N (%)	117 (100)	
Antibody status at first RTX infusion, N (%) Positive	108 (92)	
anti-dsDNA	56 (48)	
Anti-Ro	57 (49)	
Anti-La	18 (15)	
Anti-Smith	15 (13)	
Anti-Chromatin	19 (16)	
Anti-RNP	23 (20)	
Anti-Ribosomal P	6 (5)	
Anti-Cardiolipin/anti-B2-glycoprotein	14 (12)	
Prior CYC therapy, N (%)	63 (54)	
Cumulative dose of CYC, mean ± SD gram	6.6 ± 4.2	
Number of prior immunosuppressant failure (including CYC but excluding glucocorticoid), median (range)	3 (0–9)	
Concomitant antimalarials, N (%)	88 (75)	
Concomitant immunosuppressant, N (%)		
Azathioprine	19 (16)	
Methotrexate	16 (14)	
Mycophenolate Mofetil	39 (33)	
Prednisolone dose at first RTX infusion, median (IQR) mg	10 (3–20)	
ESR at first RTX infusion, median (IQR) mm/hour	29 (15–57)	
BILAG index score at baseline, N (%)		
≥1 A score	96 (82)	
No A score but ≥2 B scores	16 (14)	
BILAG domains at baseline, N (%)	Grade A	Grade B
General	9 (8)	12 (10)
Mucocutaneous	23 (20)	32 (27)
Neurological	17 (15)	17 (15)
Musculoskeletal	30 (26)	24 (20)
Cardiorespiratory	6 (5)	13 (11)
Gastrointestinal	6 (5)	0 (0)
Ophthalmic	0 (0)	0 (0)
Renal	34 (29)	0 (0)
Haematology	11 (9)	12 (10)
Global BILAG score, median (IQR)	21 (14–27)	
SLEDAI-2K score, median (IQR)	10 (6–14)	
SLICC Damage Index, median (IQR)	0 (0–1)	

ANA, antinuclear antibody; BILAG, British Isles Lupus Assessment Group; CYC, cyclophosphamide; dsDNA, double-stranded DNA; ESR, erythrocyte sedimentation rate; RNP, ribonucleic protein; RTX, rituximab; SLEDAI-2K, Systemic Lupus Erythematosus Disease Activity Index 2000; SLICC, Systemic Lupus International Collaborating Clinics (SLICC).

### Treatment characteristics

Three hundred and eighteen cycles of rituximab were administered. Median (range) duration of response in rituximab responders for cycles 1–4 (C1–4) were 52 (26–423), 52 (26—299), 57 (27–184) and 50 (29–173) weeks, respectively.

Concomitant cyclophosphamide was used in five patients who presented with life-threatening flare.

### Clinical and immunological response to first cycle rituximab

In C1, there was a good overall clinical response to rituximab. Fifty-eight (50%) patients had major clinical response, 38 (32%) partial clinical response and 21 (18%) were non-responders. The median global BILAG scores had reduced from 21 (IQR 14–27) pre-rituximab to 8 (IQR 1–10) at 6 months; p<0.001.

**Figure 1:**
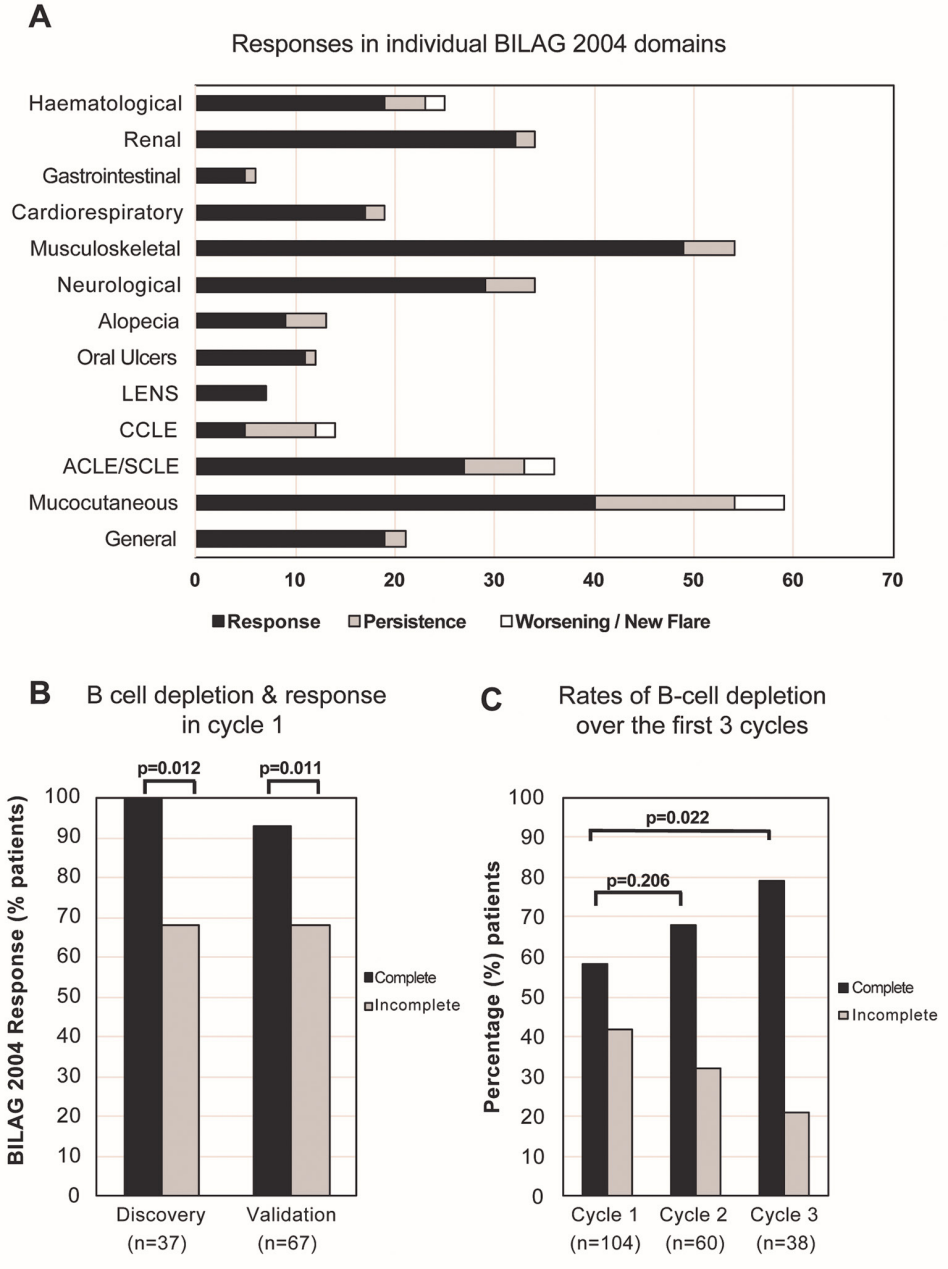
BILAG response and B-cell depletion following rituximab. (A) Majority of the individual domain improved post-rituximab although responses in the mucocutaneous and haematological domains were more varied. (B) Similar to the discovery cohort, a higher response rate was achieved in complete depletion compared with incomplete depletion groups; 93% versus 68%; p=0.011 in the validation cohort. (C) There was an incremental increase in the rates of B-cell depletion over three cycles of rituximab. ACLE, acute cutaneous lupus erythematosus; BILAG: British Isles Lupus Assessment Group; CCLE, chronic cutaneous lupus erythematosus; LENS, lupus erythematosus non-specific lesions.

Responses in individual BILAG domains are shown in figure 1A. Although majority of domains improved, responses were more variable in the mucocutaneous and haematological domains. Mucocutaneous responses to rituximab have been described in detail previously.^19^ These long-term data showed a more consistent major response in lupus erythematosus non-specific lesions and oral ulcers, while non-response in chronic cutaneous lupus erythematosus (CCLE) (CCLE vs other lupus-specific lesions; p=0.022).

The median serum anti-dsDNA titre had reduced from 109 (IQR 16–300) IU/mL pre-rituximab to 32 (IQR 7–116) IU/mL at 6 months; p<0.001. Of 46 patients with low complement (C3 and/or C4) levels pre-rituximab, levels had normalised in 25/46 (54%) at 6 months.

### Predictors of major clinical response to first cycle rituximab

Only B-cell depletion at 6 weeks increased the odds of BILAG response (major/partial) in multivariable analysis; adjusted imputed OR 13.93, 95% CI 3.11 to 62.37; p=0.001 (online supplementary table S2).

10.1136/annrheumdis-2017-211191.supp2 Supplementary material 2

As there was a high degree of response to rituximab in this cohort, we analysed predictors for major clinical response separately in order to identify patients who would respond best to therapy. In imputed univariable analysis, only younger age was associated with major response to rituximab (OR 0.97, 95% CI 0.95 to 0.99; p=0.031). While in imputed multivariable model, younger age (OR 0.97, 95% CI 0.94 to 1.00; p=0.045) and B-cell depletion at 6 weeks post-rituximab (OR 3.22, 95% CI 1.24 to 8.33; p=0.016) increased the odds of major response to rituximab (table 2).

**Table 2:** Multivariable analysis for predictors of major clinical response to first cycle rituximab

	No response/partial response n=59	Major clinical response n=58	Univariable OR (95% CI), p value (with multiple imputation)	Multivariable OR (95% CI), p value (with multiple imputation)
Age, mean (SD) years	43 (17)	37 (14)	**0.97 (0.95 to 0.99), p=0.031 per year**	**0.97 (0.94 to 1.00), p=0.045**
White, N (%)	43 (73)	37 (64)	1.53 (0.70 to 3.34), p=0.292	0.92 (0.34 to 2.47), p=0.870
Anti-dsDNA titres, mean (SD) IU/mL	147 (230)	142 (230)	1.00 (0.99 to 1.00), p=0.879 per unit	1.00 (0.99 to 1.00), p=0.632
Anti-ENA positivity, N (%)	40 (68)	38 (66)	0.91 (0.42 to 1.99), p=0.812	0.90 (0.37 to 2.22), p=0.821
Low C3 and/or C4 titres, N (%)	25 (42)	24 (41)	0.97 (0.46 to 2.04), p=0.937	1.14 (0.41 to 3.13), p=0.801
ESR, mean (SD) mm/hour*	40 (32)	41 (36)	1.00 (0.99 to 1.01), p=0.827 per unit	–
Concomitant S, N (%)†	41 (69)	35 (60)	0.67 (0.31 to 1.43), p=0.301	0.43 (0.17 to 1.09), p=0.075
Daily prednisolone dose, mean (SD) mg	13 (11)	16 (14)	1.02 (0.99 to 1.05), p=0.207 per mg	1.00 (0.97 to 1.04), p=0.713
Total BILAG score, mean (IQR)	21 (8)	24 (13)	1.03 (0.99 to 1.07), p=0.093 per point	1.02 (0.97 to 1.07), p=0.371
Total B-cell counts, mean (IQR)‡	101 (95)	138 (150)	1.00 (1.00 to 1.01), p=0.161 per unit	1.00 (1.00 to 1.01), p=0.137
B-cell depletion at 6 weeks postrituximab, N (%)	29 (49)	39 (68)	2.10 (0.95 to 4.62), p=0.065	**3.22 (1.24 to 8.33), p=0.016**

*As high collinearity was observed between ESR and total B-cell counts, only the latter was included in the multivariable analysis.

†Concomitant immunosuppressant was defined as either using methotrexate, azathioprine, mycophenolate mofetil and/or other disease modifying anti-rheumatic drugs but excluded anti-malarials.

‡count × 10^9^ cells/L) for each subset multiplied by 1000 prior to analysis.

BILAG, British Isles Lupus Assessment Group; C3/C4, complement 3 or 4; dsDNA, double-stranded DNA; ENA, extract nuclear antigen; ESR, erythrocyte sedimentation rate; IS, immunosuppressant.

### Validation of association between complete B-cell depletion and clinical response

The published discovery cohort included 37 patients with SLE.^2^ In this validation cohort, 67 subsequent and consecutive patients (with B-cell data available) were analysed. Similar to the discovery cohort, higher response rate was achieved in complete depletion compared with incomplete depletion groups (93% vs 68%; p=0.011) in this validation cohort (figure 1B).

While there was no difference at baseline, patients with complete B-cell depletion had significantly lower anti-dsDNA antibody titres at 14 weeks (p=0.030) and 26 weeks (p=0.041) versus those with incomplete depletion. In the former, C3 and C4 levels were not different at 14 weeks (p=0.064 and p=0.148, respectively) but were higher at 26 weeks (p=0.020 and p=0.022, respectively) compared with the latter group. There was no difference in anti-ENA antibodies between the two groups at 14 and 26 weeks; all p>0.10.

### Predictors for complete B-cell depletion to first cycle rituximab

Data for B-cell subsets were available for 104 (89%) patients. In imputed univariable analysis, higher anti-dsDNA titre (OR 1.00, 95% CI 0.99 to 1.00; p=0.038), normal complement levels (OR 0.41, 95% CI 0.18 to 0.91; p=0.028) and lower pre-rituximab plasmablasts (OR 0.88, 95% CI 0.80 to 0.98; p=0.015) were associated with complete B-cell depletion. While in imputed multivariable model, only normal complement levels (OR 0.29, 95% CI 0.09 to 0.90; p=0.032) and lower pre-rituximab plasmablasts (OR 0.86, 95% CI 0.78 to 0.96; p=0.007) predicted complete B-cell depletion post-rituximab (online supplementary table S4).

### B-cell depletion and associated serious infection

As most of the serious infection episodes occurred in C1 and C2 (n=23 in 15 patients), we analysed the association between complete B-cell depletion and serious infection. After two cycles, there were no difference in the serious infection rates between complete and incomplete depletion groups (8/98 (8.2%) and 7/73 (9.6%), respectively; p=0.789).

### Plasmablast repopulation as a biomarker of relapse

At 6 months, B-cells were detectable in 81% of the C1 responders. This time-point preceded all relapses. As the median of duration of response was 52 weeks, we divided the patients in this validation cohort (n=25 with B-cells data available) into two groups: (1) earlier relapse (≤12 months from first rituximab) and (2) later relapse (>12 months). A 12-month relapse time is clinically significant as it indicates that a 6-monthly retreatment may not be necessarily needed in these patients. Similar to the discovery cohort, the ROC indicated that a plasmablast count of >0.0008×10^9^/L at 6 months yielded 73% (95% CI 45% to 92%) sensitivity and 90% (95% CI 56% to 99%) specificity in predicting earlier relapse; area under the curve of 0.86 (online supplementary figure S1).

10.1136/annrheumdis-2017-211191.supp1 Supplementary Figure 1

**Figure 2:**
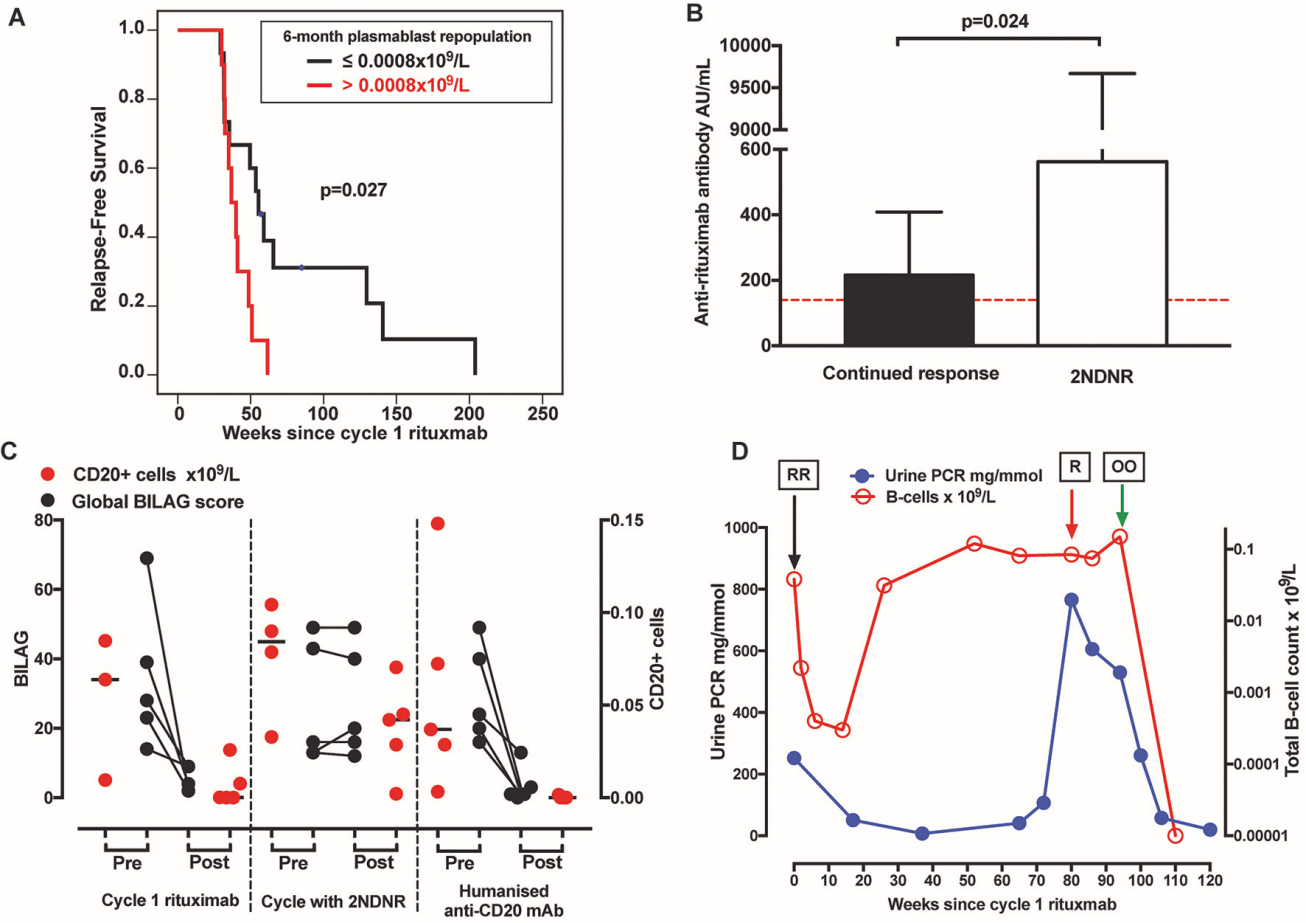
2NDNR to rituximab and efficacy of alternative humanised anti-CD20 antibodies. (A) In this validation cohort, detection of plasmablasts >0.0008×10^9^/L at 6 months predicted earlier relapse. (B) The phenomenon 2NDNR was associated with anti-rituximab antibody. The dotted red line represents normal cut-off of the test. (C) The Global BILAG score and CD20+ B-cells are plotted for each patient. The black line in the CD20+ B-cells figure represents the median. (D) An example of a case where proteinuria was normalised following a switch to ocrelizumab. ‘RR’ represents 2x infusions of rituximab, ‘R’ represents a single infusion as the patient cannot not complete the second due to severe infusion reaction and ‘OO’ represents 2x infusions of ocrelizumab. The total B-cell counts were transformed to natural log. 2NDNR, secondary non-depletion non-response; BILAG, British Isles Lupus Assessment Group.

Of the patients with plasmablasts >0.0008×10^9^/L at 6 months, relapse rates within the next 26 and 52 weeks were 90% and 100%, respectively. While of the patients with plasmablasts ≤0.0008×10^9^/L at 6 months, relapse rates within the next 26 and 52 weeks were 33% and 73%, respectively (figure 2A).

There were no differences in anti-dsDNA titres, total BILAG score and memory B-cells at 6 months between the earlier versus later relapse groups, p=0.475, p=0.985 and p=0.414, respectively.

### Retreatment of first cycle non-responders

In RA, we showed that retreatment of initial non-responders with incomplete B-cell depletion led to improved response rate in C2.^20^ Of the 21 patients who were C1 non-responders, nine were retreated with rituximab. The domains that persisted at grade A/B in C1 were mucocutaneous (n=4), musculoskeletal (n=3), renal (n=2) and haematology (n=3). After retreatment, none of these patients responded. Additionally, four patients had clinical features that were suggestive of immunogenicity.

### Retreatment of first cycle responders

**Figure 3:**
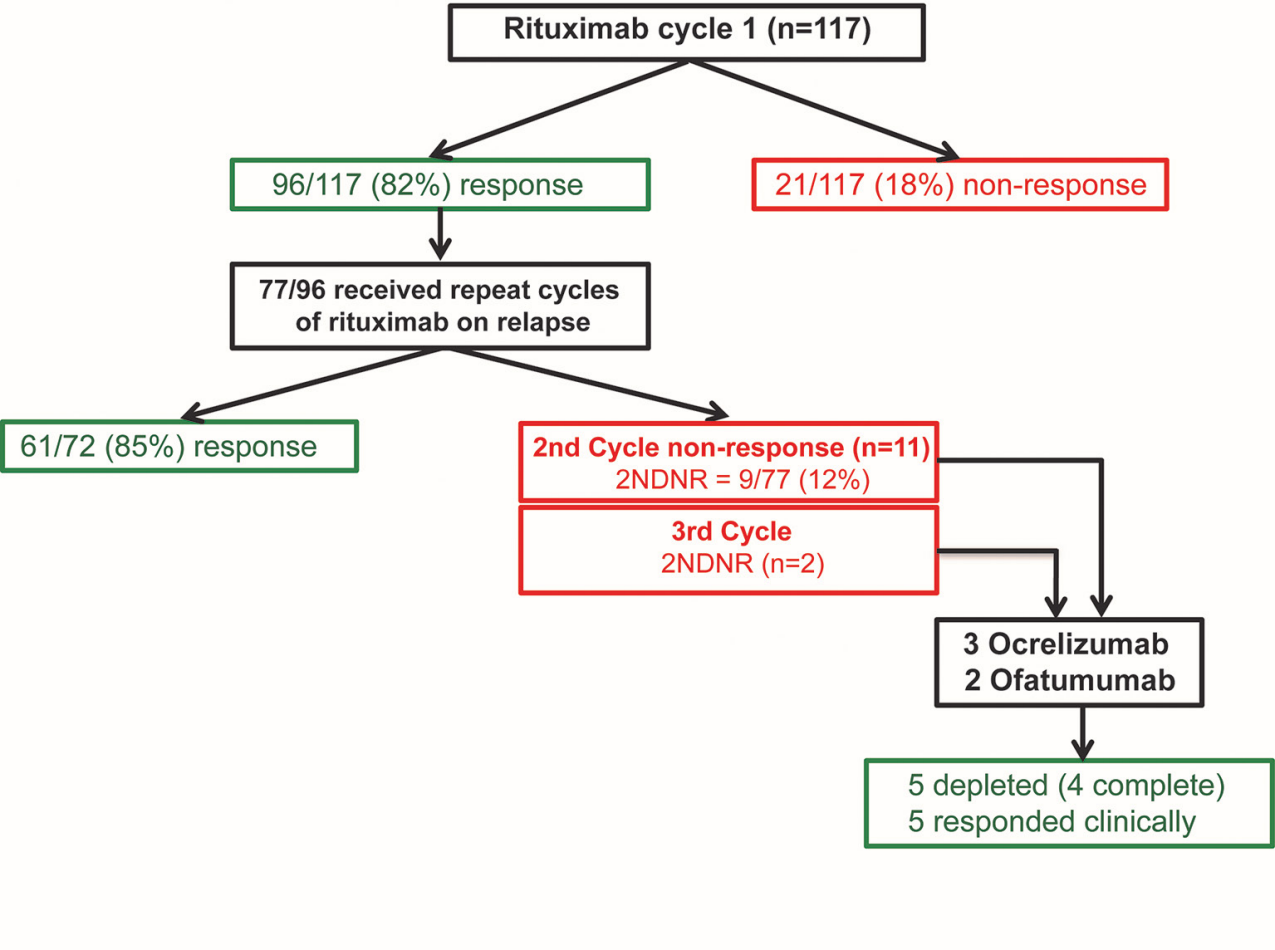
Efficacy of repeat cycles with rituximab in systemic lupus erythematosus. There was a high rate of initial clinical response to rituximab in this cohort, 96/117 (82%). Seventy-seven responders who had clinical relapse were retreated in C2. Of these, 61/72 (85%) continued to response in C2. Of the C2 non-responders, 9/11 met 2NDNR criteria. Five were switched to ocrelizumab/ofatumumab resulted in depletion and response in all. 2NDNR, secondary non-depletion and non-response; C1, cycle 1.

Of the 96 patients who were C1 responders, 77 (with complete data on 72) were retreated on clinical relapse. Of these, 61/72 (85%) responded in C2 (figure 3). Numerically higher rate of B-cell depletion was achieved in C2 compared with C1 (68% versus 58%, respectively; p=0.206) and depletion improved over subsequent cycle, C3 versus C1 (79% vs 58% respectively; p=0.022) (figure 1C).

Twelve out of 38 patients who were C1 partial responders were retreated at 6 months. Of these, major clinical response was achieved in 10/12 (83%) in C2. One patient had worsening of arthritis, while another had 2NDNR in C2.

Of the 11 patients who were C2 non-responders, nine met 2NDNR criteria. Therefore, the incidence of 2NDNR in this cohort was 9/77 (12%). In C3, another two patients had 2NDNR.

### Association of 2NDNR with antirituximab antibody

Post-rituximab sera for 5/9 patients with 2NDNR were tested for anti-rituximab antibodies. Of these, all 5/5 (100%) were tested positive. In contrast, of the 16 patients who were C2 responders, 9/16 (56%) were also tested positive for anti-rituximab antibodies. The median anti-rituximab levels were higher in the former, 562 (IQR 394–9670) AU/mL compared with the latter, 217 (IQR 0–409) AU/mL; p=0.024 (figure 2B).

### Factors associated with 2NDNR

Risk factors for 2NDNR were lack of concomitant immunosuppressant (p=0.023) and higher pre-rituximab plasmablasts (p<0.001) (table 3). Concomitant corticosteroid dose, duration of response in C1, clinical response category in C1, pre-rituximab global BILAG score, pre-rituximab naïve and memory B-cells were not associated with 2NDNR; all p>0.10.

**Table 3:** Factors associated with secondary non-depletion non-response to rituximab (2NDNR)

Characteristics prior to rituximab retreatment	Continued to respond (n=61)	2NDNR (n=9)	p Value
Concomitant IS, N (%)	41 (67)	2 (22)	**0.023**
Prednisolone, median (IQR) mg	5 (0–10)	5 (0–17.5)	0.729
Duration of response, median (IQR) weeks	50 (36–107)	62 (52–164)	0.239
Total BILAG score, median (IQR)	16 (12–21)	24 (12–27)	0.209
Partial clinical response in cycle 1, N (%)	24 (39)	3 (33)	0.731
Naïve B-cells, median (IQR) 10^9^ cells/L	0.0349 (0.0071–0.0735)	0.0620 (0.0101–0.0950)	0.296
Memory B-cells, median (IQR) x 10^9^/L	0.0019 (0.0010–0.0047)	0.0090 (0.0054–0.0394)	0.175
Plasmablasts, median (IQR) x 10^9^/L	0.0011 (0.0004–0.0036)	0.0086 (0.0052–0.0227)	**<0.001**

*NDNR, secondary non-depletion and non-response; IS, immunosuppressant.

### Efficacy of switching to humanised anti-CD20 antibodies

Following 2NDNR, treatment for five patients were switched to humanised anti-CD20 mAbs (3=ocrelizumab and 2=ofatumumab). Post-treatment, complete depletion of CD20+ cells were achieved in 4/5 patients, while the remaining one had substantially low counts (0.0016×10^9^/L).

The median global BILAG scores had reduced from 24 (IQR 18–45) pre-treatment to 1 (IQR 0–8) post-treatment; p=0.008 (figure 2C). The individual BILAG response is shown in figure 2D and described in online supplementary table S5. One patient with class IV-G (active with moderate scarring) who had progressed into end-stage renal failure was treated with ofatumumab, mainly for severe thrombocytopaenia with a view for renal transplantation preparation. Post-treatment, her platelet had normalised from 45×10^9^/L (pre-treatment), renal parameters were stable and she successfully underwent live donor renal transplantation.

## Discussion

The clinical challenges for the use of rituximab in SLE include defining subgroups of patients likely to respond to the initial and subsequent cycles and optimal repeat treatment strategy. By capturing data of all patients with SLE who were treated with rituximab in this largest reported cohort, as well as long-term follow-up, this study offers insights into pragmatic use of rituximab and has implications for the future development of targeted therapies.

In this study, the only consistent predictor of any (and major) clinical response to rituximab is B-cell depletion (as measured using HSFC) at 6 weeks post-rituximab, which we have now validated in an independent cohort. This underlines the immunomodulatory action of rituximab in correcting autoimmune B-cell function and normalising autoantibody titres and complement levels without increasing the risk of severe infection. From treatment stratification perspective, our data support the rationale for B-cell monitoring during therapy. Thus, prior to rituximab, by assessing patients for low complement levels and higher plasmablasts, treatment modification can be employed to improve depletion, either by increasing the dose or adding an extra infusion, as we previously showed in RA.^21^ At 6 weeks post-rituximab, complete depletion is a marker of good response to therapy. For those with incomplete depletion, close monitoring is required. At 6 months post-rituximab, repopulation of plasmablasts of >0.0008×10^9^/L increases the risk of clinical relapse within the following 6 months. Therefore, these patients can be considered for early retreatment in order to reduce the higher burden of B-cell numbers and enhance depletion in the subsequent cycle. Importantly, for those with plasmablasts of ≤0.0008×10^9^/L at 6 months, monitoring for clinical relapse would appear an acceptable strategy.

Regardless of response, about 12% subsequently developed 2NDNR in C2. This phenomenon is associated with rituximab anti-drug antibodies. However, measuring anti-rituximab antibody alone is not enough to identify patients as 2NDNR as over half of the patients who were tested positive responded in that particular cycle. Instead, clinical features, that is, severe infusion reaction and non-response and measuring B-cells, are more meaningful. Lack of concomitant oral immunosuppressant and higher pre-rituximab plasmablasts predicted 2NDNR. Oral immunosuppressant use was decided at physician discretion, but our data suggest they might have a role in preventing immunogenicity. The exact mechanism for the association with plasmablast number is unknown, but plasmablasts are markers for overall B-cell activation. Following initial depletion with rituximab, B-cell-activating factor levels increase and promote the formation of plasmablasts.^22^ This early increase in plasmablasts enhances the formation of follicular T-helper cells, thus creating a positive feedback loop that perpetuates antibody-driven inflammation and may explain why some patients become refractory to rituximab in SLE.^23^

Following 2NDNR to rituximab, switching to humanised anti-CD20 mAbs restores depletion and response in SLE. Ocrelizumab and ofatumumab are both type 1 anti-CD20 mAbs. The primary endpoint was met in ocrelizumab-treated groups in RA trials^24^ and was investigated in SLE.^25^ However, development in these indications was halted after an increase in opportunistic infections, some of which fatal were reported.^26^ All three patients in our study had major clinical responses and prolonged remission for over 5-year period post-ocrelizumab. Ofatumumab is licenced for resistant chronic lymphocytic leukaemia and has demonstrated efficacy in RA.^27^ Both patients in our study responded well to ofatumumab included one who achieved complete depletion for the first time from B-cell depleting therapy. Additionally, a few case series have recently reported on its efficacy in extrarenal and refractory lupus nephritis.^28 29^ Alternatively, other anti-CD20 agents with enhanced antibody-dependent cellular cytotoxicity may be more effective in SLE. *In vitro* obinutuzumab demonstrated enhanced depletion was achieved with this type 2 mAb, compared with rituximab.^30^

This study has several limitations. First, an interobserver variability could have occurred in BILAG assessments due to the lengthy follow-up duration and a cohort that was highly heterogeneous in lupus manifestations. However, the BILAG scores reflected the clinician’s intention-to-treat, and the patients were managed in a dedicated single centre, thus allowing for consistency in assessment. Second, B-cells and laboratory data were missing in some cases. As these were deemed missing at random, multiple imputation was used to reduce potential bias in parameter estimation as well as enhancing generalisability of the results. Next, concomitant therapy with immunosuppressant were used in more than 60% of the patients, thus efficacy could not be attributed to rituximab alone. Lastly, the lack of control group limits interpretation of efficacy and safety of rituximab.

In conclusion, treatment with anti-CD20 agents can be guided by B-cell monitoring with the aim of achieving complete depletion. About one in eight patients with SLE lose depletion on repeat cycles of rituximab regardless of prior response and secondary non-depletion is associated with anti-rituximab antibodies. Concomitant oral immunosuppressant may help to prevent this. If 2NDNR occurs, switching to humanised anti-CD20 mAbs restores depletion and response. Therefore, alternative anti-CD20 antibodies may be more consistently effective in SLE treatment and several ongoing trials are addressing these issues.
