# Supplementary material for: Predicting and managing primary and secondary non-response to rituximab using B-cell biomarkers in systemic lupus erythematosus
Source: Ann Rheum Dis. 2017 Jul 6;76(11):1829–36. doi: 10.1136/annrheumdis-2017-211191 (PMC5705851; doi:10.1136/annrheumdis-2017-211191)
Supplement: Supplementary material 2 [file annrheumdis-2017-211191supp002.docx]

**SUPPLEMENTARY DATA**

**Predicting and managing primary and secondary non-response to rituximab using B-cell biomarkers in systemic lupus erythematosus**

**CONTENTS:**

1. Table S1: Responses in individual BILAG domains at 26 weeks post-rituximab
2. Table S2: Multivariable analysis for predictors of clinical response (major/partial) to rituximab
3. Table S3: Validation of B-cell depletion and clinical response in first cycle rituximab
4. Table S4: Multivariable analysis for predictors of B-cell depletion at 6 weeks post-rituximab
5. Figure S1: Plasmablast as a biomarker for relapse and secondary non-depletion prediction
6. Table S5: Efficacy of alternative humanised anti-CD20 agents in patients with 2NDNR
7. S6: Long-term safety of B-cell depletion therapy
8. Table S6: Serious adverse events
9. Table S7: IgG levels measured at 6 months of each cycle of rituximab infusion

**Table S1: Responses in individual BILAG domains at 26 weeks post-rituximab (cycle 1)**

| **Domains** | **No cases at baseline (Grade A/B)** | **MCR** | **PCR** | **Severe Persistence (Grade A)** | **Moderate Persistence (Grade B)** | **Worsening** | **New Flare** |
| --- | --- | --- | --- | --- | --- | --- | --- |
| General | 21 (9/12) | 19 (90) | 0 | 2 (10) | 0 | 0 | 0 |
| Mucocutaneous | 55 (23/32) | 35 (64) | 5 (9) | 3 (5) | 11 (20) | 2 (4)* | 3 (5) |
| ACLE/SCLE | 34 (10/24) | 24 (71) | 3 (9) | 2 (6) | 4 (12) | 2 (6)* | 1 (3) |
| CCLE | 12 (5/7) | 3 (25) | 2 (17) | 1 (8) | 6 (50) | 0 | 2 (17) |
| LENS | 7 (5/2) | 7 (100) | 0 | 0 | 0 | 0 | 0 |
| Oral ulcers | 12 (2/10) | 11 (92) | 0 | 0 | 1 (8) | 0 | 0 |
| Alopecia | 13 (0/13) | 9 (69) | 0 | 0 | 4 (31) | 0 | 0 |
| Neurological | 34 (17/17) | 22 (65) | 7 (21) | 0 | 5 (14) | 0 | 0 |
| Musculoskeletal | 54 (30/24) | 41 (76) | 8 (15) | 4 (7) | 1 (2) | 0 | 0 |
| Cardiorespiratory | 19 (6/13) | 17 (89) | 0 | 0 | 2 (11) | 0 | 0 |
| Gastrointestinal | 6 (6/0) | 5 (83) | 0 | 0 | 1 (17) | 0 | 0 |
| Ophthalmic | 0 | 0 | 0 | 0 | 0 | 0 | 0 |
| Renal | 34 (34/0) | 24 (71) | 8 (24) | 2 (5) | 0 | 0 | 0 |
| Haematology | 23 (11/12) | 17 (73) | 2 (9) | 2 (9) | 2 (9) | 2 (9) | 0 |

ACLE: Acute cutaneous lupus erythematosus; CCLE: Chronic cutaneous lupus erythematosus, LENS: Lupus erythematosus non-specific lesions, MCR: major clinical response, PCR: Partial clinical response

* One patient with BILAG B for ACLE rash pre-rituximab had worsening of psoriasiform lesions (BILAG A) at 6 months post-rituximab. Hence, the total percentage for MCR, PCR, severe and moderate persistence in the mucocutaneous and ACLE/SCLE domains did not add up to 100%

**Table S2: Multivariable analysis for predictors of clinical response (major/partial) to rituximab**

|  | **No Response**  **n=21** | **Major/Partial Clinical Response**  **n=96** | **Univariable OR (95% CI),P-value**  **(with multiple imputation)** | **Multivariable OR (95% CI),**  **P-value**  **(with multiple imputation)** |
| --- | --- | --- | --- | --- |
| Age, mean (SD) years | 39 (14) | 40 (16) | 1.00 (0.97-1.03), p=0.833 per year | 0.99 (0.95-1.04), p=0.785 |
| White, N (%) | 14 (67) | 66 (69) | 0.91 (0.33-2.48), p=0.852 | 0.56 (0.13-2.44), p=0.440 |
| Anti-dsDNA titres, mean (SD) IU/ml | 121 (217) | 149 (233) | 1.00 (0.99-1.00), p=0.464 per unit | 1.00 (0.99-1.00), p=0.243 |
| Anti-ENA positivity, N (%) | 19 (90) | 60 (62) | **0.18 (0.04-0.84), p=0.028** | 0.21 (0.04-1.21), p=0.080 |
| Low C3 and/or C4 titres, N (%)* | 8 (38) | 42 (44) | 1.26 (0.48-3.33), p=0.640 | - |
| ESR, mean (SD) mm/hour | 45 (33) | 39 (34) | 1.00 (0.98-1.01), p=0.618 per unit | 0.99 (0.97-1.01), p=0.525 |
| Concomitant IS, N (%)** | 14 (67) | 62 (65) | 0.91 (0.34-2.47), p=0.856 | - |
| Daily Prednisolone dose, mean (SD) mg | 16 (13) | 14 (13) | 0.98 (0.95-1.02), p=0.390 per mg | 0.98 (0.93-1.03), p=0.511 |
| Total BILAG score, mean (IQR) | 21 (11) | 23 (11) | 1.02 (0.97-1.07), p=0.486 per point | 1.02 (0.96-1.09), p=0.487 |
| Total B-cell counts, mean (IQR)*** | 132 (103) | 116 (128) | 1.00 (0.99-1.00), p=0.727 per unit | 1.00 (0.99-1.01), p=0.986 |
| B-cell depletion at 6 weeks post-rituximab, N (%) | 3 (14) | 65 (68) | **11.07 (2.97-41.20), p<0.001** | **13.93 (3.11-62.37), p=0.001** |

BILAG: British Isles Lupus Assessment Group, C3/C4: Complement 3 or 4, dsDNA: Double stranded deoxyribonucleic acid, ENA: Extract nuclear antigen, ESR: Erythrocyte sedimentation rate, IQR: Interquartile range, IS: Immunosuppressant, OR: Odds ratio, SD: Standard deviation

* As there were high collinearity between concomitant IS and B-cell depletion, low complement and Total B-cell counts and concomitant IS and low complement, the last two variables were excluded in the multivariable analysis

* * Concomitant immunosuppressant was defined as either using methotrexate, azathioprine, mycophenolate mofetil and/or other disease modifying anti-rheumatic drugs but excluded anti-malarials

***(count x 10^9^ cells/L) for each subset multiply by 1000 prior to analysis

**Table S3: Validation of B-cell depletion and clinical response in first cycle rituximab**

| **Cohort** | **Depletion** | **Non Response** | **Response** | **p-value** |
| --- | --- | --- | --- | --- |
| **Discovery (n=37)** | Incomplete | 8/22 (36%) | 14/22 (64%) | 0.012 |
|  | Complete | 0/15 (0%) | 15/15 (100%) |  |
| **Validation (n=67)** | Incomplete | 7/22 (32%) | 15/22 (68%) | 0.011 |
|  | Complete | 3/45 (7%) | 42/45 (93%) |  |
| **Combined (n=104)** | Incomplete | 15/44 (34%) | 29/44 (66%) | <0.001 |
|  | Complete | 3/60 (5%) | 57/60 (95%) |  |

**Table S4: Multivariable analysis for predictors of B-cell depletion at 6 weeks post-rituximab**

|  | **Incomplete B-cell Depletion**  **n=44** | **Complete B-cell Depletion**  **n=60** | **Univariable OR (95% CI),**  **P-value**  **(with multiple imputation)** | **Multivariable OR (95% CI),**  **P-value**  **(with multiple imputation)** |
| --- | --- | --- | --- | --- |
| Anti-dsDNA titres, mean (SD) IU/ml | 200 (282) | 101 (166) | **1.00 (0.99-1.00), p=0.038** | 1.00 (0.99-1.00), p=0.105 |
| Anti-ENA positivity, N (%) | 31 (70) | 36 (60) | 0.63 (0.27-1.44), p=0.273 | 0.75 (0.27-2.07), p=0.579 |
| Low C3 and/or C4 titres, N (%) | 25 (57) | 21 (35) | **0.41 (0.18-0.91), p=0.028** | **0.29 (0.09-0.90), p=0.032** |
| Concomitant IS, N (%) | 25 (57) | 43 (72) | 1.92 (0.85-4.36), p=0.118 | 2.66 (0.98-7.27), p=0.055 |
| Corticosteroid dose, mean (SD) mg | 15 (11) | 13 (14) | 0.99 (0.96-1.02), p=0.339 | 1.02 (0.97-1.06), p=0.429 |
| Total BILAG score, mean (IQR) | 23 (11) | 23 (11) | 1.00 (0.97-1.04), p=0.938 per point | 1.05 (1.00-1.11), p=0.064 |
| Naïve B-cell counts, mean (SD)* | 101 (105) | 74 (79) | 0.99 (0.99-1.00), p=0.202 | 0.99 (0.99-1.00), p=0.111 |
| Memory B-cell counts, mean (SD)* | 29 (31) | 27 (70) | 1.00 (0.99-1.01), p=0.889 | 1.00 (0.99-1.01), p=0.658 |
| Plasmablast counts, mean (SD)* | 8 (10) | 3 (5) | **0.88 (0.80-0.98), p=0.015** | **0.86 (0.78-0.96), p=0.007** |

BILAG: British Isles Lupus Assessment Group, C3/C4: Complement 3 or 4, dsDNA: Double stranded deoxyribonucleic acid, ENA: Extract nuclear antigen, IQR: Interquartile range, IS: Immunosuppressant, OR: Odds ratio, SD: Standard deviation

*(count x 10^9^ cells/L) for each subset multiply by 1000 prior to analysis

**LEGEND TO FIGURE**

**Figure S1: Plasmablast as a biomarker of relapse and secondary non-depletion prediction.** A) Higher pre-rituximab plasmblasts were associated with secondary non-depletion non-response to cycle 2 of rituximab. Data are presented as box plots, where the top of the boxes represent the median and the line bars represent the 25th to 75th percentiles. B) Receiver operator curve indicated that a plasmablast count of >0.0008 x 10^9^/L at 6 months demonstrated 73% sensitivity and 90% specificity in predicting earlier relapse.

**Table S5: Efficacy of alternative humanised anti-CD20 agents in patients with 2NDNR**

| **Patient ID** | **Biologics** | **Pre-Treatment BILAG Activity** | **Pre-Treatment Global BILAG score** | **Post-Treatment BILAG Activity** | **Post-Treatment Global BILAG score** | **Pre-Treatment CD20+ B-cells x 10 ^9^/L** | **Post-Treatment CD20+ B-cells x 10 ^9^/L** |
| --- | --- | --- | --- | --- | --- | --- | --- |
| 01 | OCR | B – General  A –Mucocutaneous  B – Musculoskeletal  A – renal | 40 | D – General  C – Mucocutaneous  D – Musculoskeletal  D - Renal | 1 | 0.0032 | 0 |
| 02 | OCR | B – General  A – Renal | 20 | D – General  D - Renal | 0 | 0.1481 | 0 |
| 03 | OCR | B – Neurological  B – Musculoskeletal | 16 | D – Neurological  C - Musculoskeletal | 1 | 0.0724 | 0.0016 |
| 04 | OFA | A – Renal  A – Haematology | 24 | B – Renal  C - Haematology | 13 | 0.0369 | 0 |
| 05 | OFA | B – General  A – Mucocutaneous  B – Neurological  B – Musculoskeletal  A – Renal  C – Haematology | 49 | D – General  C – Mucocutaneous  D – Neurological  D – Musculoskeletal  C – Renal  C - Haematology | 3 | 0.0286 | 0 |

BILAG: British Isles Lupus Assessment Group, OCR: Ocrelizumab, Ofa: Ofatumumab

* 1 patient with severe SLE who had incomplete B-cell depletion and non-responder in C1 was retreated with rituximab but subsequently developed immunogenicity. She was treated with ocrelizumab resulted in enhanced depletion, biological response ie: normalisation of anti-dsDNA and complement levels as well as was able to be discharged home (after 3 months of prolonged hospitalisation). Unfortunately she died 5 months later due to multi-organ failure. This patient was not included in the above as she did not meet 2NDNR criteria.

**S6 Long-term Safety of B-cell Depletion Therapy in SLE**

**Method:**

Safety assessments which included severe adverse events (SAEs) and serious infection were recorded irrespective of possible association with SLE and or therapy. SAEs were defined as those resulting in either hospitalisation that lasted more than 24 hours, flares requiring intravenous therapy, malignancies, life-threatening situations or death. Data for serious infections was gathered from hospital admission records using Patient Access Centre (PAS) system and was later confirmed with case notes.

**Results:**

Hundred and thirty-eight SAEs were recorded in 54 patients who were treated with rituximab. Of these, 130 were hospitalisation episodes (median duration 5 (IQR 3-9) days), 3 malignancies and 5 deaths (online supplementary Table S6). The causes of deaths were intracranial/subarachnoid haemorrhage=2, pneumonia=1, urinary sepsis=1 and multi-organ failure=1. Thirty-three serious infections (6.7/100 patient-years) were recorded in 23 patients, mostly due to chest infection (n=15). There were 5 opportunistic infections recorded were mycobacterium avium complex=1, pneumocystis jiroveci pneumonia=1 in a patient who was simultaneously diagnosed as having HIV infection, cytomegalovirus=1, disseminated varicella zoster=1 and disseminated candidiasis. 36% (n=12) and 64% (n=21) of the serious infections occurred within 3 and 6 months respectively from the last rituximab infusion in any cycle. No cases of progressive multi-focal leukoencephalopathy (PML) was observed.

As most of the serious infection episodes occurred in C1 and C2 of rituximab (n=23 in 15 patients), we analysed the association between complete B-cell depletion and serious infection. After two cycles, there was no difference in the rates of serious infection between complete and incomplete depletion groups; 8/98 (8.2%) and 7/73 (9.6%) respectively; p=0.789.

In C1, there was a reduction in the mean IgG at 6 months compared to baseline, 14.57 (SD 7.42) g/L versus 12.06 (4.73) g/L, mean difference -2.52 (95% CI -4.23 to -0.80) g/L; p<0.004. After 4 cycles of therapy, repeat cycles of rituximab on clinical relapse resulted in progressive deterioration in IgG level compared to baseline; p<0.001 after adjusting for Bonferroni correction (online supplementary Table S7). Although the reduction in immunoglobulin may represent immunological response to rituximab, immunoglobulin levels need to be monitored closely during therapy. Prior to first rituximab, 5 patients had low IgG (normal 6.0-16.0 g/L). Post-rituximab, only 5 patients developed new low IgG after this prolonged follow-up. Thus, the incidence and prevalence of low IgG in this cohort was 4.4% and 8.5% respectively. 4/10 (40%) of patients with low IgG had serious infections. While 2 (2%) patients required concomitant immunoglobulin replacement due to recurrent infection-associated with low IgG.

Low IgM (normal 0.60-2.50 g/L) following rituximab was more common than low IgG. The prevalence of the former in C1 and C2 was 41/117 (35%). However, in both cycles, low IgM was not associated with serious infection; p=0.283.

Six SAEs were recorded in 3 patients who were treated with ocrelizumab. Of these, 4 were serious infections (14/100 patient-years) that included one opportunistic infection with cytomegalovirus. One death occurred in a patient with severe SLE who died of multi-organ failure at 5 months post-ocrelizumab. No serious infection was recorded in the two patients who were treated with ofatumumab.

**Table S6: Serious Adverse Events**

|  | **Rituximab**  **(n=117)** | **Ocrelizumab**  **(n=4)** | **Ofatumumab**  **(n=2)** |
| --- | --- | --- | --- |
| All severe adverse events  No. of severe adverse events  No. patients with severe adverse events (%) | 138  54 (46) | 5  3 (75) | 5  1 (50) |
| All serious infection, no. events  Pneumonia  Urinary tract infection  Opportunistic infections  Cellulitis/Skin abscess  Intra-abdominal abscess  Infectious diarrhoea  Necrotising fasciitis  Line infection | 33  15  7  4  3  1  1  1  1 | 3  1  0  1  0  1  0  0  0 | 0  0  0  0  0  0  0  0  0 |
| All other hospitalisation, no. events  SLE flare  Infusion reaction/serum sickness  Viral illness  Acute kidney injury  Bowel surgery/Hernia repair  Thromboembolism  Diverticulitis/Perforated colon  Orthopaedics surgery (Elective)  Kidney transplant  Acute coronary syndrome/Angiogram  Intracerebral haemorrhage  Palpitation/Atrial fibrillation  Avascular necrosis  Seizure  Kidney stones  Other medical | 97  31  12  8  5  5  4  4  3  3  2  2  2  1  1  0  14 | 1  0  0  0  0  0  0  0  0  0  0  0  0  0  0  1  0 | 5  0  0  0  3  0  0  0  0  1  0  0  0  0  1  0  0 |
| All malignancy, no. events  Renal transitional cell carcinoma  Thymoma  Uterine carcinoma | 3  1  1  1 | 0 | 0 |
| Deaths | 5 | 1 | 0 |

**Table S7: IgG levels measured at 6 months of each cycle of rituximab infusion**

| **Time point** | **Patients**  **Treated, N** | **Mean IgG**  **(SD) g/L** | **p-value (versus**  **previous cycle)*** | **Patients with low IgG, N (%)**** | **Patients with new low IgG since previous cycle, N (%)** |
| --- | --- | --- | --- | --- | --- |
| Baseline | 117 | 14.57 (7.42) | N/A | 5/108 (5) | 5/108 (5) |
| Cycle 1 6 months | 117 | 12.06 (4.73) | 0.004 | 6/101(6) | 3/101 (3) |
| Cycle 2 6 months | 77 | 11.88 (5.76) | 0.823 | 4/72 (6) | 2/72 (3) |
| Cycle 3 6 months | 47 | 9.86 (3.65) | 0.058 | 3/36 (8) | 0 (0) |
| Cycle 4 6 months | 28 | 9.10 (3.50) | 0.437 | 3/22 (14) | 0 (0) |

* p value was calculated using paired T-test

** values show number of patients with data available

Multiple comparisons between IgG levels measured at 6 months of each cycle to baseline IgG showed overall p<0.001 using one-way analysis of variance (ANOVA) adjusting for Bonferroni correction
