# Supplementary figures and images for: Predicting and managing primary and secondary non-response to rituximab using B-cell biomarkers in systemic lupus erythematosus
Source: Ann Rheum Dis. 2017 Jul 6;76(11):1829–36. doi: 10.1136/annrheumdis-2017-211191 (PMC5705851; doi:10.1136/annrheumdis-2017-211191)

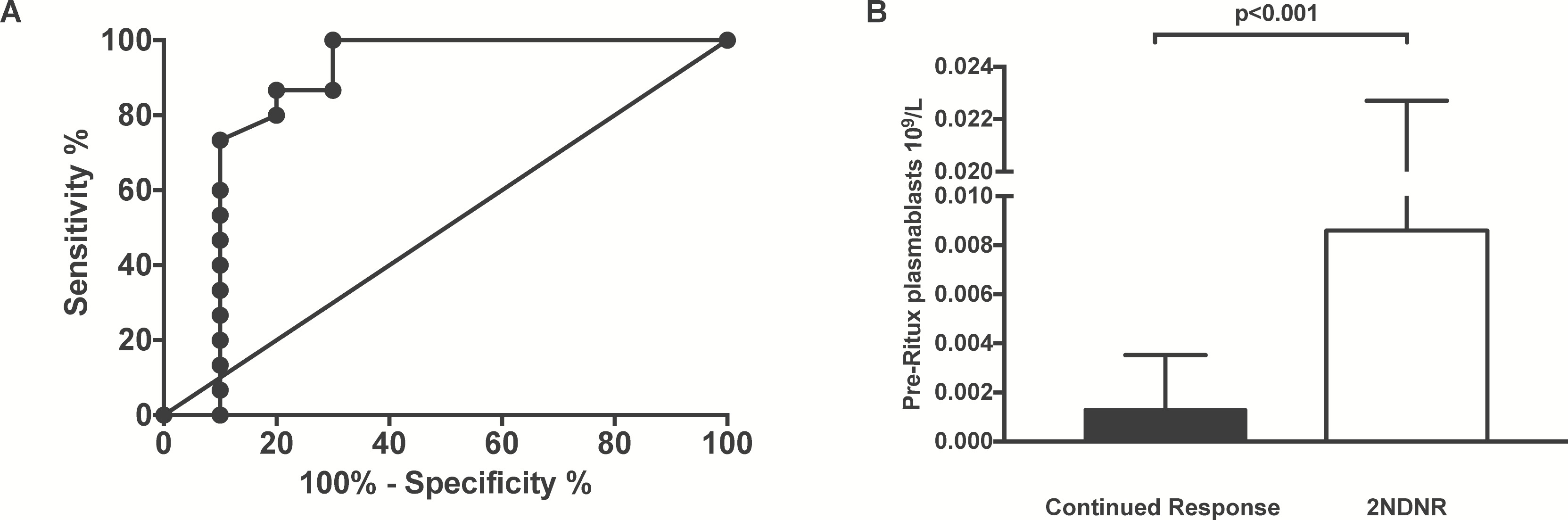

Supplement: Supplementary Figure 1 [file annrheumdis-2017-211191supp001.jpg]
